# Hydrogen peroxide diffusion and scavenging shapes mitochondrial network instability and failure by sensitizing ROS-induced ROS release

**DOI:** 10.1038/s41598-020-71308-z

**Published:** 2020-09-25

**Authors:** Brent Millare, Brian O’Rourke, Natalia Trayanova

**Affiliations:** 1 Department of Biomedical Engineering, Johns Hopkins University, Baltimore, 21218 USA; 2 Division of Cardiology, Department of Medicine, The Johns Hopkins University School of Medicine, Baltimore, 21205 USA

**Keywords:** Mitochondria, Cardiovascular biology

## Abstract

The mitochondrial network of cardiac cells is finely tuned for ATP delivery to sites of energy demand; however, emergent phenomena, such as mitochondrial transmembrane potential oscillations or propagating waves of depolarization have been observed under metabolic stress. While regenerative signaling by reactive oxygen species (ROS)-induced ROS release (RIRR) has been suggested as a potential trigger, it is unknown how it could lead to widespread responses. Here, we present a novel computational model of RIRR transmission that explains the mechanisms of this phenomenon. The results reveal that superoxide mediates neighbor-neighbor activation of energy-dissipating ion channels, while hydrogen peroxide distributes oxidative stress to sensitize the network to mitochondrial criticality. The findings demonstrate the feasibility of RIRR as a synchronizing factor across the dimensions of the adult heart cell and illustrate how a cascade of failures at the organellar level can scale to impact cell and organ level functions of the heart.

## Introduction

The organization of the mitochondrial network plays an important role in the normal functioning of the adult ventricular myocyte^1–3^. In order to meet the high energy demands of contraction and ion homeostasis, mitochondria in cardiomyocytes are organized in a dense regular lattice proximal to both the myofilaments and the Ca^2+^ release apparatus^4,5^. This topological arrangement can also support propagation of mitochondrial membrane potential ($\Delta \Psi_{{\text{m}}}$) changes over large distances, which has been proposed to occur either through local diffusion of signaling molecules, such as Ca^2+6^ or reactive oxygen species (ROS)^7–9^, or by direct physical connections between mitochondria^10,11^. Since communication networks comprising strongly coupled nodes and high connectivity are prone to cascading failures^12^, the mitochondrial networks of ventricular myocytes are also inherently vulnerable to such failures. These complex spatiotemporal aspects of mitochondrial organization have consequences, as they can scale to affect myocyte electrical and contractile function^9,13^, organ level emergent phenomena, such as arrhythmias^14^, or even the sudden death of the organism^15,16^.

**Figure 1:**
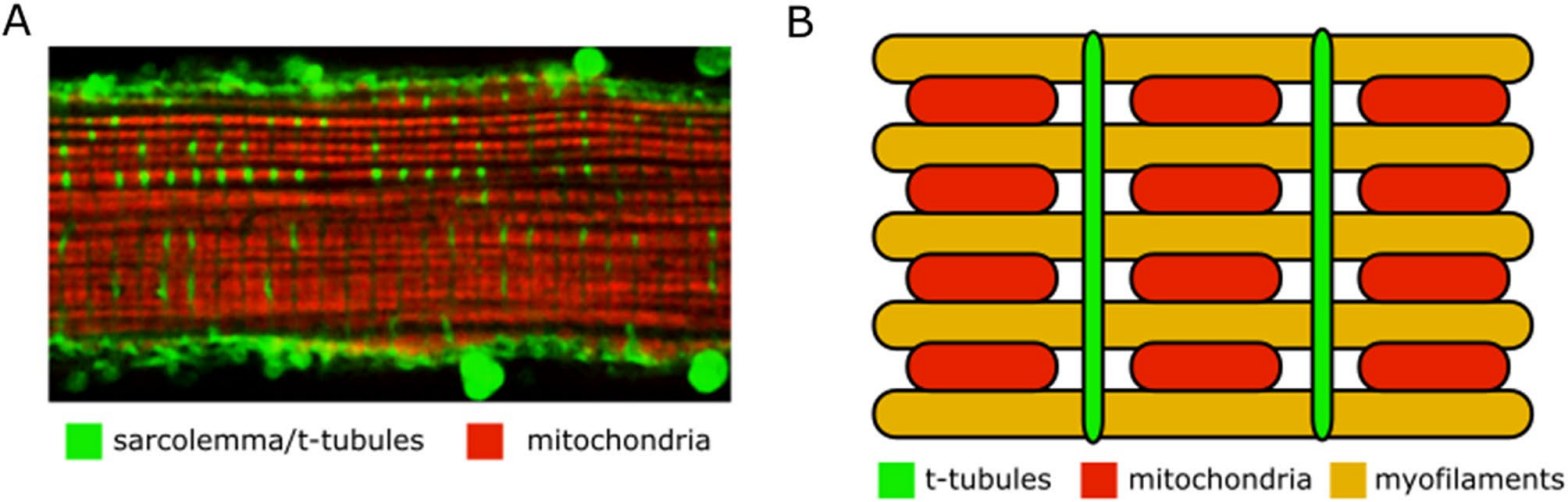
Mitochondrial arrangement in a myocyte. (**a**) Example confocal microscopy image of canine cardiomyocyte with green stained sarcolemma and t-tubules, and red stained mitochondria. Adult canine cardiomyocyte image was obtained using a 60 × lens on a two-photon laser-scanning microscope with a Ti–sapphire mode-locked laser at 780 nm. Cell was stained with tetrarhodamine methyl ester (TMRM) to visualize polarized mitochondria (red image), as previously described^9^, and surface membranes were stained with Thioflavin S (green image) to visualize t-tubules. A bandpass filters at 605 ± 25 nm was used to collect the TMRM emission and a cutoff filter was used to collect light < 490 nm for Thioflavine S. (**b**) Zoomed in schematic of mitochondrial arrangement with myofilaments colored yellow.

Mitochondrial failure can manifest as $\Delta \Psi_{{\text{m}}}$ depolarizations in the intact heart^17–19^ or in isolated cardiomyocytes subjected to oxidative or metabolic stress^7–9,20–22^. These depolarizations are observed when the mitochondrial ROS scavenging system responsible for removing excess superoxide (SO) and hydrogen peroxide (H_2_O_2_) is overwhelmed, either by excess ROS production or by limited antioxidant replenishment capacity^23,24^. Accumulation of SO near a mitochondrion is thought to trigger the opening of SO-sensitive mitochondrial inner membrane anion channels (IMAC)^25^ or sensitize other ROS-sensitive channels to opening, such as the permeability transition pore (PTP)^8,26^. Opening of such channels generates and releases additional SO from the mitochondrial matrix, resulting in $\Delta \Psi_{{\text{m}}}$ depolarization. This triggering and release process has been termed ROS-induced ROS release (RIRR)^8^. Further, when the mitochondrial network is oxidatively stressed and the antioxidant capacity is depleted, RIRR can propagate throughout the network^27^. Propagation occurs as SO released from one mitochondrion diffuses to a neighboring mitochondrion and initiates additional IMAC openings and release of accumulated SO. Superoxide dismutase (SOD) or SOD mimetics^8,9^ mitigate RIRR by facilitating the rapid conversion of SO into H_2_O_2_, which is subsequently removed by peroxidases in the cytoplasm and mitochondrial matrix. Network instability (failure) due to this mechanism is often characterized by regions of mitochondria with synchronized oscillating $\Delta \Psi_{{\text{m}}}$. Mitochondrial SO release resulting in propagation of $\Delta \Psi_{{\text{m}}}$ depolarizations has been studied in experimental and computational models^27,28^. However, the mechanisms underlying $\Delta \Psi_{{\text{m}}}$ propagation and synchronization within the mitochondrial network remain poorly understood. Furthermore, the extent and strength of coupling of network elements across the length of the heart cell (100–150 µm) is unclear. Recently, the idea that mitochondria may be electrically coupled^10^ across large cellular distances has been reintroduced^11^; however, this model is incompatible with a number of experimental observations. For example, local perturbations of $\Delta \Psi_{{\text{m}}}$ propagate through cardiac cells only after a long delay (minutes) between a local stimulus and the cell-wide $\Delta \Psi_{{\text{m}}}$ response^9^; spatial clusters of $\Delta \Psi_{{\text{m}}}$ oscillation dynamically evolve over time and are modulated by substrate availability^29–32^; and highly localized spontaneous oscillations in $\Delta \Psi_{{\text{m}}}$ can occur in small clusters^7^ or individual mitochondria^20,21^ without propagating to neighboring mitochondria. In the adult heart cell, mitochondria are physically separated laterally, by intervening myofilaments, and longitudinally, by t-tubules at each z-line (Fig. 1), hence, it remains to be determined if physical connections contribute to cell-wide propagating or synchronized phenomena.

Synchronization of the mitochondrial network by diffusible messengers would require a mechanism that account for both short- and long-range interactions among mitochondria. A key question is whether short-range mitochondrial neighbor-neighbor interactions that depend on messengers with a brief half-life, such as SO, can scale across the cell. In this study, we propose a novel mechanism, that the sensitivity to local SO-induced RIRR depends on the longer range effects of H_2_O_2_, which has a longer lifetime than SO and can more easily diffuse throughout the cytoplasm. We show, by developing a novel computational model of the mitochondrial network, that H_2_O_2_ diffusion underlies the transmission of mitochondrial network failure by distributing oxidative stress and subsequently depleting ROS scavenging capacity, thus sensitizing mitochondria across the network to RIRR. This mechanism can explain “mitochondrial criticality” in cardiac cells^2^, when the network becomes ultrasensitive to cell-wide ∆Ψ_m_ depolarization or oscillations subsequent to a build-up of oxidative stress in a majority fraction of the network. The model provides a deeper understanding of the mechanisms of mitochondrial network failure associated with cardiac pathophysiologies such as ischemia/reperfusion injury^14^ or sudden death associated with chronic heart failure^16^.

## Results

### Computational model of the mitochondrial network

**Figure 2:**
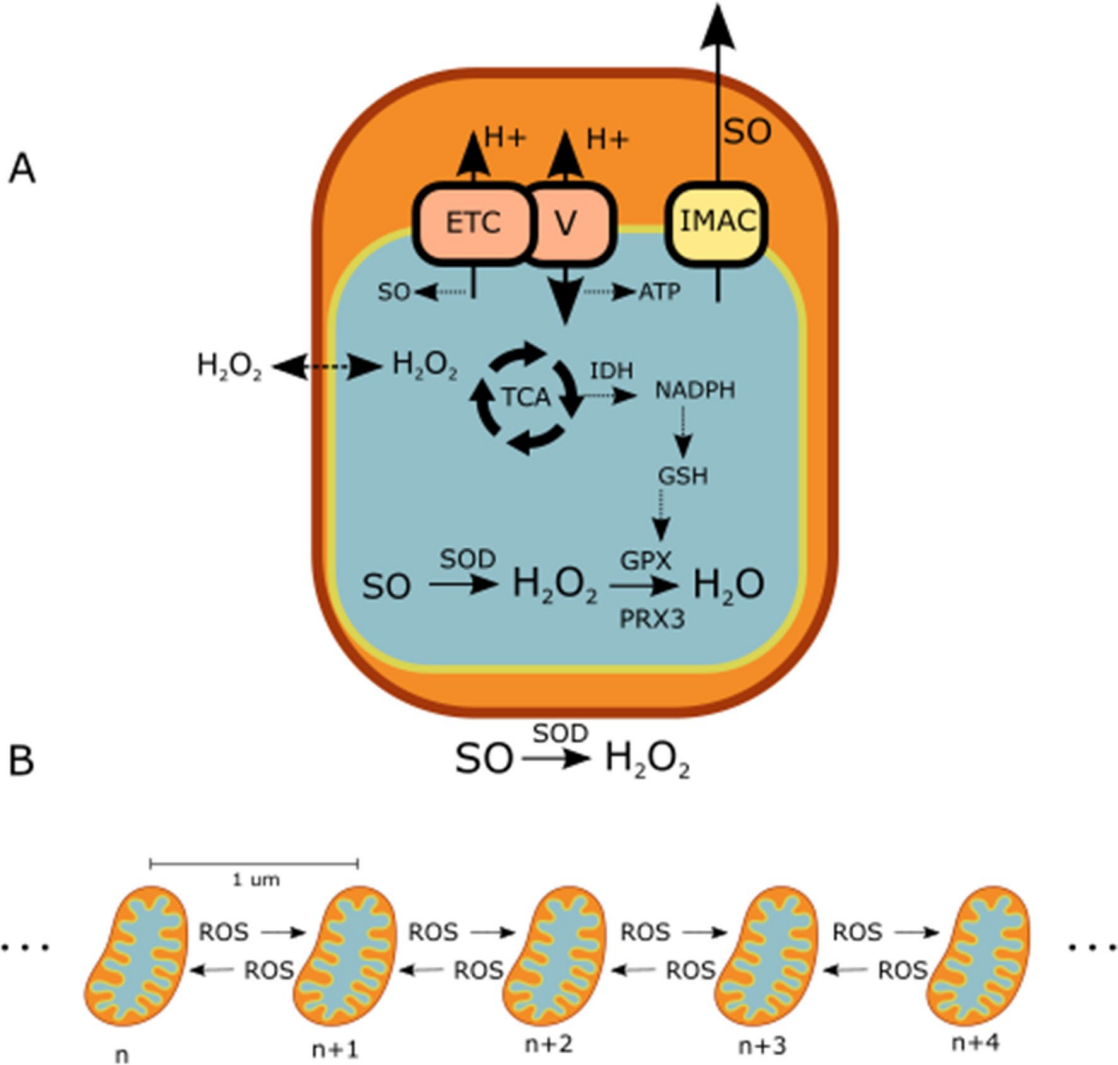
Model Schematics. (**a**) Single mitochondrial model: TCA cycle, ROS scavenging, ROS transport via IMAC, and SO production from the electron transport chain. (**b**) 1-dimensional mitochondrial network model: ROS can diffuse between neighboring mitochondria.

In this study, we developed a novel computational model of the mitochondrial network in the cardiac myocyte. The model (Fig. 2) includes a detailed representation of the ROS scavenging system, ROS diffusion between mitochondria, and ROS production. We represent the ROS production within the mitochondrial matrix that is from intrinsic origins and from externally induced alterations. H_2_O_2_ diffusion between mitochondria was represented to enable H_2_O_2_ to act as a communicator of oxidative stress across the network (Fig. 2b). We regionally stimulated the network with ROS, as described in Methods, and observed the network behavior with and without H_2_O_2_ diffusion.

### Mitochondrial network response to ROS stimulus

**Figure 3:**
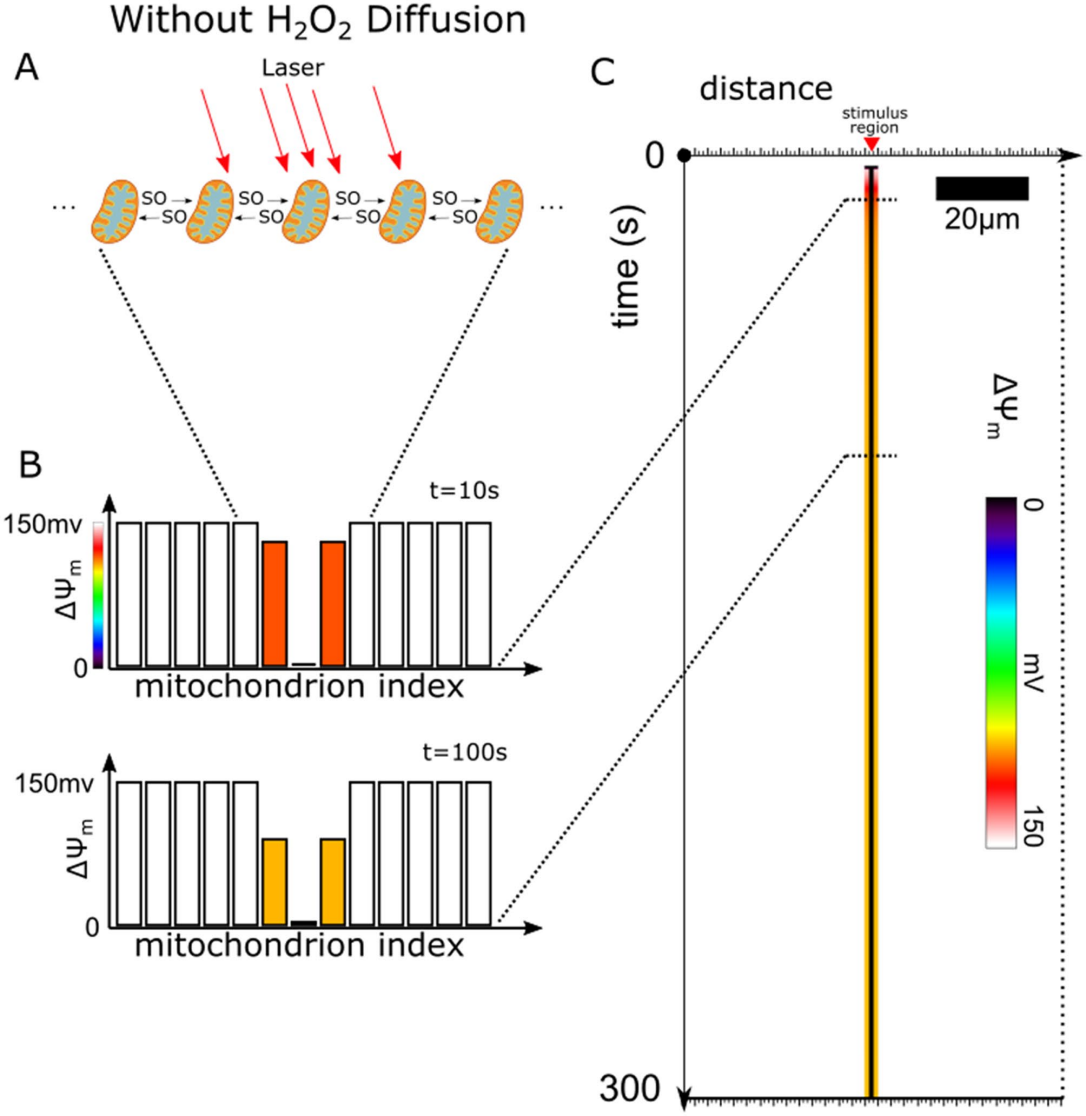
ROS Stimulated Mitochondrial Network Without H_2_O_2_ Diffusion. (**a**) Schematic of the mitochondrial network with a region of mitochondria stimulated to produce ROS over time and with only SO diffusion between mitochondria. The red arrows pointing at the mitochondria represent the laser stimulation. The central mitochondrion has the largest density of arrows and representing the largest SO_stim_ of 5 × 10^−5^ mM ms^−1^. The other two mitochondria are on the edge of the laser stimulation region, thus receive less laser stimulation, and have a lower SO_stim_ of 1.4 × 10^−5^ mM ms^−1^. (**b**) Bar plots of the $\Delta \Psi_{{\text{m}}}$ of mitochondria in the network at 2 different time points, 10 and 100 s. (**c**) Time-line plot of evolution of $\Delta \Psi_{{\text{m}}}$ of the mitochondria in the network. The plot presents a 2D representation of the time evolution of the 1D mitochondrial network, where color represents the $\Delta \Psi_{{\text{m}}}$ value. The horizontal axis represents the spatial extent of the 1D mitochondrial network, and the vertical axis represents time. The white space in the plot represents the mitochondria at spans of time where they are polarized and have a normal membrane potential around 150 mV. Mitochondria that depolarized had a reduced membrane potential and are represented by the different colors depending on depolarization magnitude. The central mitochondrion, which is permanently depolarized, is represented as a black vertical line in this plot.

Figure 3 presents the results of simulations without H_2_O_2_ diffusion. At time 0, all the mitochondria were polarized with a $\Delta \Psi_{{\text{m}}}$ around 150 mV. After 3 s, the central stimulated mitochondrion (Fig. 3a) depolarized. Interestingly, it depolarized substantially (> 95% reduction in $\Delta \Psi_{{\text{m}}}$), while the stimulated mitochondria at the edge of the region (the immediate neighbors of the central mitochondrion) only partially depolarized to 115 mV after 10 s, and only a bit further to 105 mV after 100 s (Fig. 3b). The remaining mitochondria in the network did not depolarize for the simulation duration of 300 s (Fig. 3c). Figure 3c shows the evolution of the membrane potential of each mitochondrion in the network.

**Figure 4:**
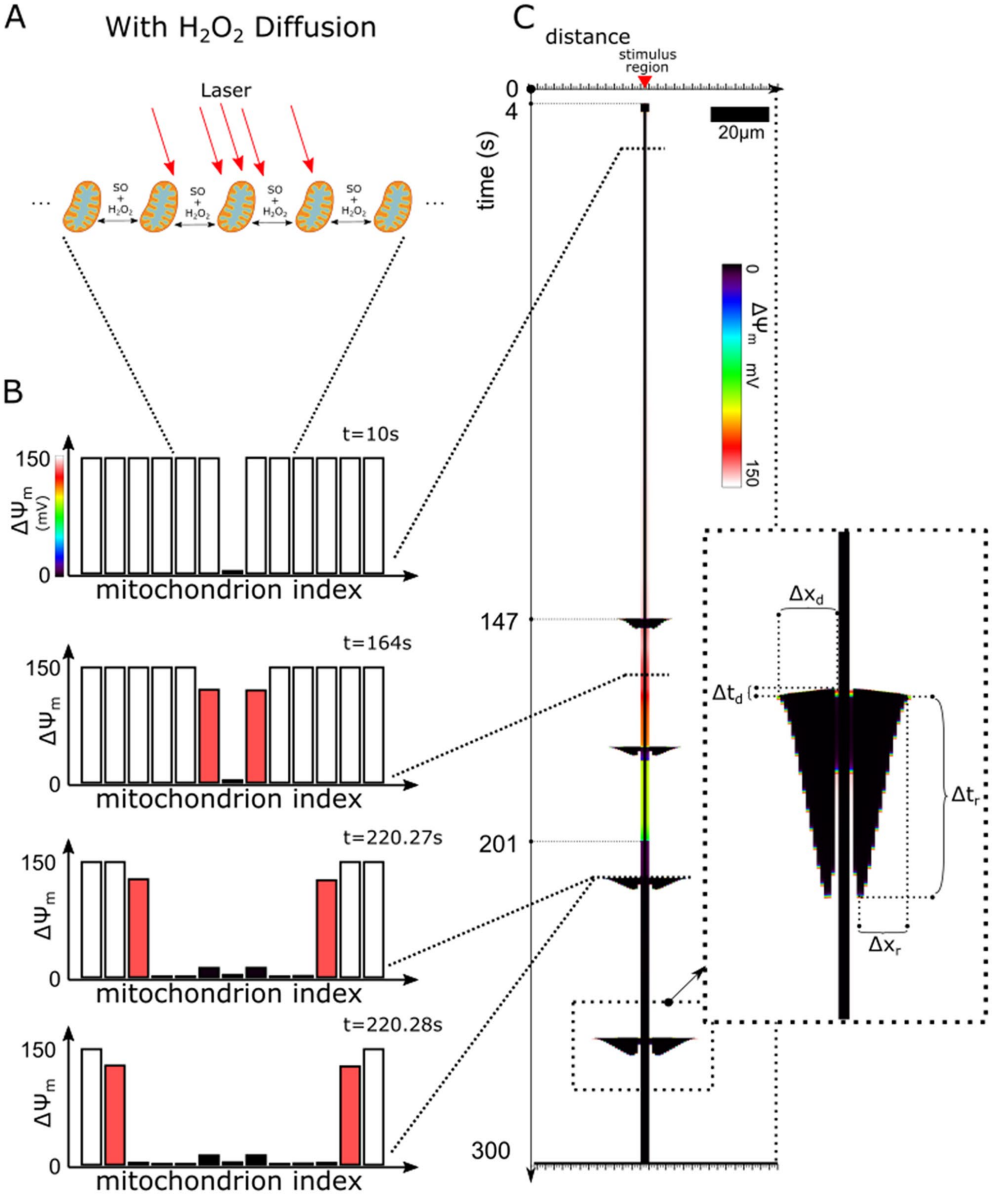
ROS Stimulated Mitochondrial Network with H_2_O_2_ diffusion. (**a**) Schematic of mitochondrial network with a region of mitochondria stimulated to produce ROS over time and with both SO and H_2_O_2_ diffusion between mitochondria. (**b**) Bar plots of the $\Delta \Psi_{{\text{m}}}$ of mitochondria in the network at different time points. (**c**) Time-line plot of evolution of $\Delta \Psi_{{\text{m}}}$ of the mitochondria in the network. The inset displays a zoomed in view of the last propagating depolarization and repolarization pattern. The propagating depolarization pattern's duration is marked by dashed line $\Delta$t_d_ and the pattern's length is marked by the dashed line marked $\Delta$x_d_. Similarly, the repolarization pattern's duration and length are also marked by the dashed lines $\Delta$t_r_ and $\Delta$x_r_ respectively.

Figure 4 presents the simulations with H_2_O_2_ diffusion. Like before, the stimulation protocol involved 3 mitochondria in the center of the network with elevated SO_stim_ (Fig. 4a). Unlike the simulations without H_2_O_2_ diffusion, multiple mitochondria depolarized (Fig. 4b). First, at time 0, all mitochondria were polarized. Next, the 3 stimulated mitochondria depolarized after 4 s. The middle mitochondrion, which had the largest SO generation rate, permanently depolarized. The behavior of this mitochondrion matched the behavior found in experiments, where laser flash stimulated mitochondria irreversibly depolarized after a few seconds^2,9^. In contrast to the central mitochondrion, the surrounding two mitochondria were stimulated to a lesser degree and exhibited sustained depolarizations only 201 s after the laser flash. At 4 s in the simulation, these two mitochondria first underwent a transient depolarization lasting 2.5 s. Afterwards, the two mitochondria displayed partial depolarizations. Unlike the results of the simulations without H_2_O_2_ diffusion, these mitochondria continued to gradually depolarize and eventually fully depolarized after 201 s. Thus, the response of the 3 stimulated mitochondria is represented by the thick vertical black line in Fig. 4c. Collectively, these simulation results reproduced the experimental observations where mitochondria gradually irreversibly depolarized within the laser flash stimulated region^2,9^.

The black horizontal extrusions starting at 147 s in Fig. 4c represent the mitochondrial depolarizations that propagated outward from the stimulated mitochondria. This delay in the response of the bulk of the network to oxidative stress has been consistently observed in both laser flash stimulated mitochondrial networks and chemically stressed mitochondrial networks (The paper by Zhou et al. demonstrated such behavior in their supplemental video (S3))^27^. In our simulation results, the extent of the propagating depolarizations (Fig. 4c inset length $\Delta$x_r_ and $\Delta$x_d_) gradually increased after each consecutive depolarization wave (Fig. 4c). This network behavior is consistent with behavior in the stimulation experiments where depolarization propagations did not, at first, extend globally throughout the network. The depolarization waves in our simulations repeated roughly every 30 s, which was within the range of normal frequencies found in experiments. Each of these outward propagating depolarization waves were then followed by inwardly-directed repolarization waves. This last-in/first-out depolarization/repolarization pattern exhibited a compressed diamond like shape (Fig. 4c). Our simulation results reproduced the bias in faster depolarization propagation velocity versus the repolarization propagation velocity (Fig. 4c inset ($\Delta$x_d_/$\Delta$t_d_):($\Delta$x_r_/$\Delta$t_r_)). The total time frame of the depolarization wave and repolarization wave took 3 s at the first oscillation, and increased to 5 s for the last observed oscillation. This trend of increasing the average depolarization duration of the mitochondria in these depolarization waves was also observed in experiments^2,9,27^.

### Mechanisms driving mitochondrial network failure patterns

**Figure 5:**
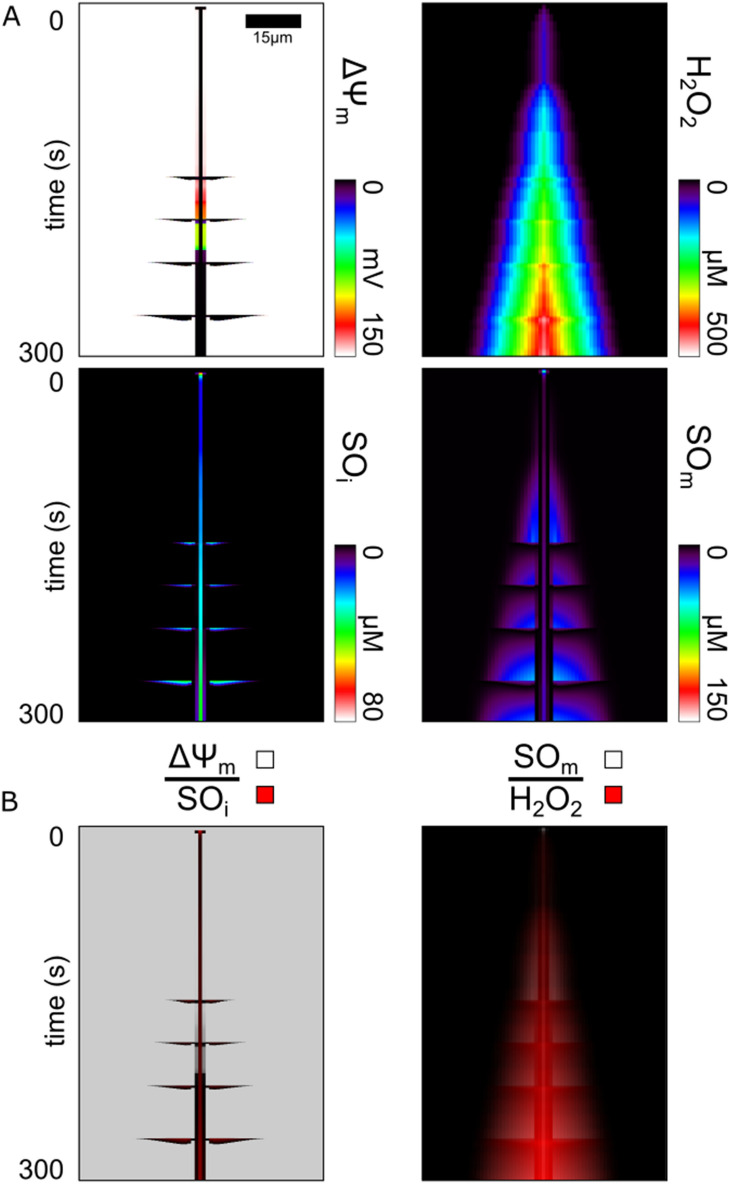
Evolution of the Region Vulnerable to Failure and Regions of ROS Accumulation in the Mitochondrial Network. (**a**) From the same simulation run shown in Fig. 4, shown are the plots of mitochondrial membrane potential ($\Delta \Psi_{{\text{m}}}$), hydrogen peroxide (H_2_O_2_), inter-mitochondrial superoxide (SO_i_), and matrix superoxide (SO_m_). (**b**) Pairs of time-line plots from (**a**) created using a partially transparent overlay to highlight encasement or overlap of accumulation regions. The top overlayed plot uses varying transparent shades of white to a maximum of 80% opaque to represent different quantities specified above the line. Likewise, the bottom plot uses varying transparent shades of red to a maximum of 100% opaque to represent different concentrations of the species below the line.

From the simulation with H_2_O_2_ diffusion, Fig. 5a displays the evolution of $\Delta \Psi_{{\text{m}}}$ alongside the evolution of ROS accumulation. We found that the mitochondrial network accumulated H_2_O_2_ around the stimulus location (Fig. 5a H_2_O_2_). The H_2_O_2_ concentration monotonically increased in the stimulus region over time for the entire simulation duration (300 s). The center mitochondrion had an average H_2_O_2_ accumulation rate of 1.7µM/s. The immediate neighboring mitochondria had a similar average H_2_O_2_ accumulation rate but only after a delay of 3–5 s, and remained lower in concentration by 25 µM compared with the stimulus region. This pattern of delay and reduced H_2_O_2_ concentration was repeated for successive neighboring mitochondria with roughly the same step decrease of 25 µM per mitochondrion. This pattern ended when the next successive neighboring H_2_O_2_ concentration reached the baseline (< ~ 10 nM) and thus marked the boundary of the extent of H_2_O_2_ accumulation. A roughly linear H_2_O_2_ gradient formed, extending from the stimulus location to this boundary. Altogether, both the size of the H_2_O_2_ accumulation region and the H_2_O_2_ of the mitochondria within the region increased over time.

In contrast, the patterns of SO accumulation did not form a simple linear gradient over time and instead displayed complex fluctuating patterns both along the string of mitochondria (i.e. horizontal direction in Fig. 5a) and over time (vertically in Fig. 5a), both in the matrix and the inter-mitochondrial space (SO_m_ & SO_i_). The laser flash stimulated mitochondria begins generating ROS at the start of the simulation and as a result, the matrix SO of the central mitochondrion quickly increased to above 150uM. At this concentration, the SO leakage into the intermembrane space triggered the central mitochondrion's depolarization at 4 s. The central mitochondrion then released more SO into the intermitochondrial space, reducing its matrix SO to 13.6uM. This SO concentration remained relatively stable, and only increased at a rate of 113 nM/s as long as the oxidative stress stimulus was maintained, reaching an observed maximum of 45 µM after 300 s. Interestingly, besides the initial release of SO at the onset of the central mitochondrion's depolarization, the mitochondria directly adjacent to the central mitochondrion exhibited low matrix SO (< 1 µM) compared to its outer neighbors for the first 221 s of simulation. These mitochondria displayed partial depolarizations, where only a slight reduction of $\Delta \Psi_{{\text{m}}}$ (from 151 to 148 mV) occurred. These partial depolarizations were caused by the partial conductance increase of IMAC, which was caused by SO stimulation that diffused from the central mitochondrion. SO generated in these partially stimulated mitochondria continuously escaped and prevented matrix SO accumulation. The generated SO instead transferred to the intermitochondrial space, raising the concentration of SO in that space to ~ 50 nM, which was substantially larger than the < 1 nM of the more distant polarized neighbors, but not large enough to induce positive feedback of RIRR bursting. The remaining mitochondria in the network displayed matrix SO accumulation patterns similar to their H_2_O_2_ accumulation patterns, but only prior to their next depolarization event. Also, during this period before the next depolarization, intermitochondrial SO did not accumulate. Only the region of mitochondria with enough accumulated matrix SO (> 1 nM) depolarized. Sufficient accumulation of matrix SO was required such that a triggered release of matrix SO into the intermitochondrial space would induce further self-release, which then needed to be large enough to trigger depolarizations in neighboring mitochondria. This transport process resulted in a rapid transient increase in intermitochondrial SO depending on the amount of accumulated stored SO, and also a rapid decrease in matrix SO. After sufficient SO scavenging, the depolarized mitochondria repolarized once the intermitochondrial SO returned to baseline levels. These patterns of matrix SO accumulation and release continued after each subsequent depolarization propagation. Further, the length of the extent of the accumulation region increased with each cycle where the additional number of mitochondria recruited per depolarization ranged between 2 and 4. Interestingly, the extent of matrix SO accumulation region was contained within the extent of the H_2_O_2_ accumulation region and the extent of intermitochondrial SO accumulation was contained within the depolarized region (Fig. 5b).

**Figure 6:**
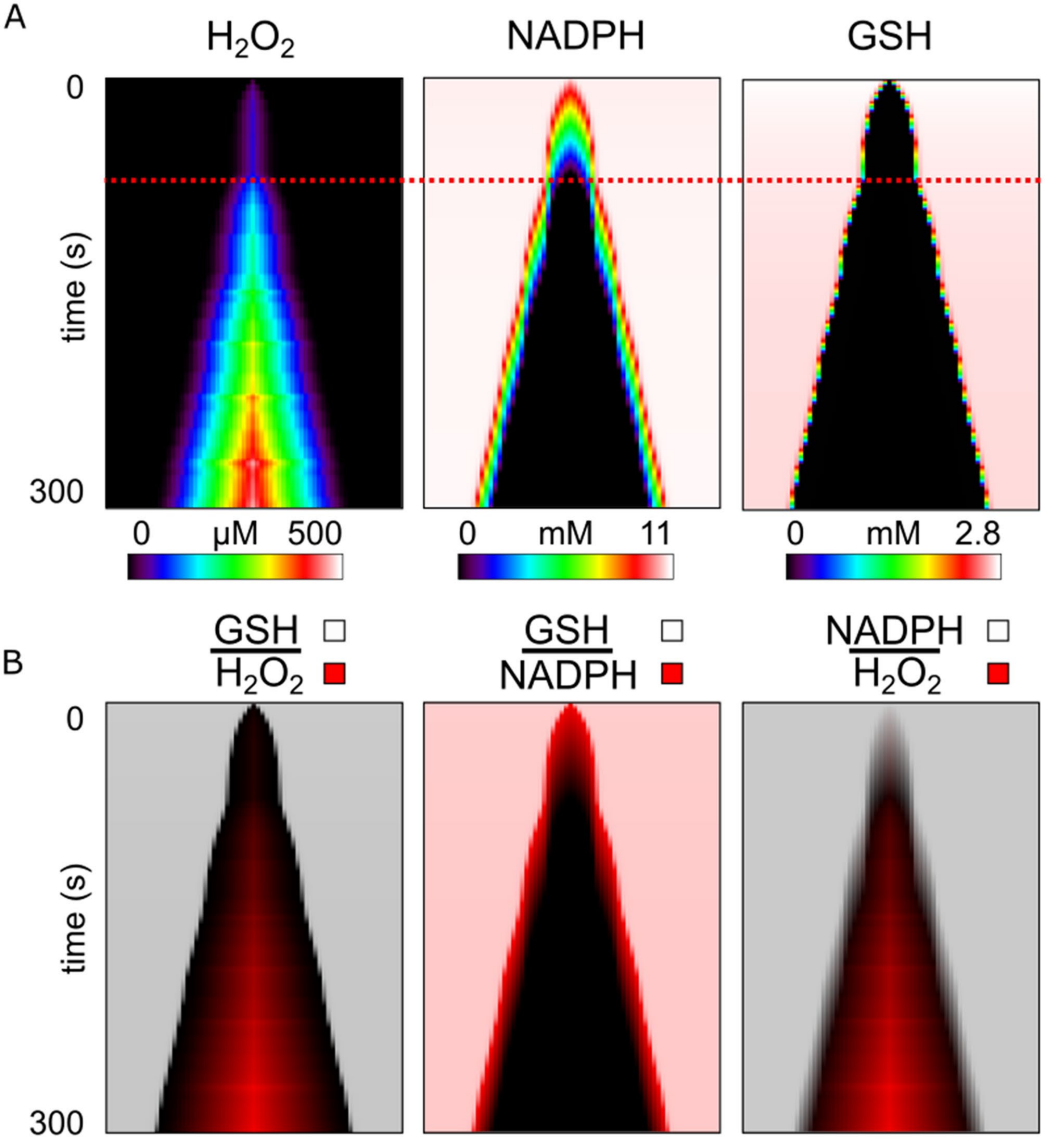
Evolution of H_2_O_2_ Accumulation and H_2_O_2_ Scavenging Capacity Depletion in the Mitochondrial Network. (**a**) From the same simulation run shown in Figs. 4 and 5, show are the plots over time of hydrogen peroxide (H_2_O_2_), glutathione (GSH) which is the energetic driver of the H_2_O_2_ scavenger enzyme glutathione peroxidase, and nicotinamide adenine dinucleotide phosphate (NADPH) which is the energetic driver of the GSH producing enzyme glutathione reductase. The red dashed line marks the time when the respective extent of accumulation or depletion resumes outward growth towards the rest of the network after a ~ 10 s hiatus. (**b**) Pairs of time-line plots from (**a**) created using a partially transparent overlay to highlight encasement or overlap of accumulation and depletion regions. The top overlayed plot uses varying transparent shades of white to a maximum of 80% opaque to represent different concentrations of the species listed above the line. Likewise, the bottom plot uses varying transparent shades of red to a maximum of 100% opaque to represent different concentrations of the species below the line.

The H_2_O_2_ scavenging system component GSH formed a depletion zone (Fig. 6a GSH) that enveloped the H_2_O_2_ accumulation region (Fig. 6b GSH|H_2_O_2_). NADPH, the energetic supplier to that system, also formed a depletion zone but was instead enveloped by the GSH depletion region (Fig. 6b GSH | NADPH). Interestingly, the growth of the GSH depletion zone underwent two phases. First, a depletion zone of ~ 12 um formed at the center over the first 40 s while NADPH was elevated. After the NADPH of that region depleted, the second phase of GSH depletion occurred and the GSH and NADPH depletion region gradually increased in extent over time. This change in extent growth is denoted by the dashed red line in Fig. 6a. Thus, depletion of the cascaded energetic drivers of the H_2_O_2_ scavenging system was required for initiating H_2_O_2_ accumulation and for further increasing the extent of this H_2_O_2_ accumulation region.

### Dependence of mitochondrial network response on ROS stimulus makeup

**Figure 7:**
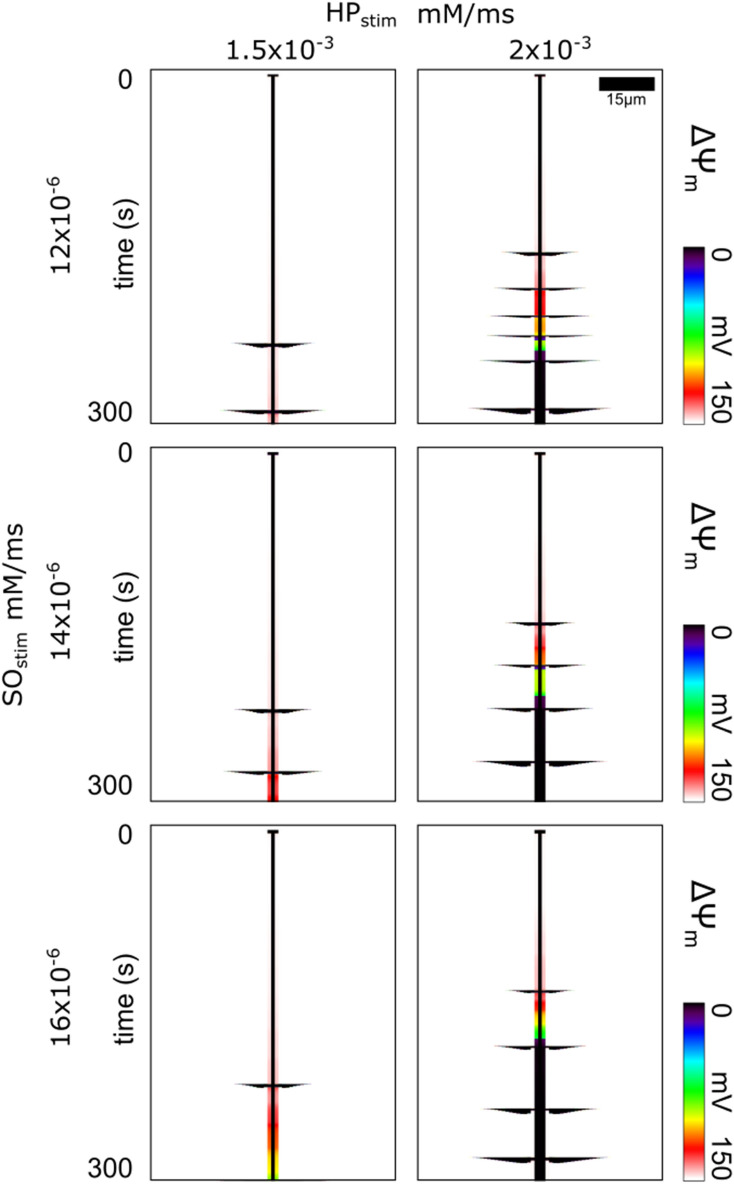
Variations in Mitochondrial Network Oscillation Frequency and Timing Depending on the ROS Stimulus Makeup. Time-line plots of $\Delta \Psi_{{\text{m}}}$ of the mitochondria in the network for different HP_stim_ and SO_stim_ rates. Propagating depolarization waves of varying frequency and extent are visible in each of the plots depending on the ROS stimulus rates.

Figure 7 presents the results of simulations where the HP_stim_ and SO_stim_ rates were varied independently to compare different ratios of H_2_O_2_ and SO generation rates. Like before, the HP_stim_ was only assigned to the central laser flash stimulated mitochondrion. The different SO_stim_ rates were assigned to the immediate neighboring stimulated mitochondria since the central mitochondrion's SO_stim_ was defined by depolarization status protocol like described before. The case shown in Fig. 7 second row & second column involved the same stimulation parameters as in Fig. 4 where SO_stim_ was 1.4 × 10^–5^ and HP_stim_ was 2 × 10^–3^. Reducing HP_stim_ to 1.5 × 10^–3^ resulted in the $\Delta \Psi_{{\text{m}}}$ patterns presented in Fig. 7 second row & first column. Reducing HP_stim_ from 2 × 10^–3^ to 1.5 × 10^–3^ increased the delay of the first propagating depolarization by ~ 77 s, decreased the number of observed propagating depolarizations by over half, increased the time between depolarizations by ~ 30 s, and decreased the size of the extent of depolarizing mitochondria by ~ 30%. The start times for all propagating depolarization waves in each plot were different for each ROS combination (Table 1). No propagating depolarization waves were observed when HP_stim_ was reduced to 1 × 10^–3^. Increasing SO_stim_ decreased the delay in the first propagating depolarization wave by ~ 8 s. This decrease was explained by the increased rate of matrix SO accumulation of the stimulated mitochondria, which causes increased leak to the intermitochondrial space sooner, which causes intermitochondrial SO to reach the IMAC opening trigger threshold sooner. This additional flux into the intermitochondrial space can also reduce the matrix SO accumulation of neighbors while their neighbor's matrix SO levels are still low. The additional intermitochondrial SO slightly increases their neighbor's IMAC conductance that leaks out some matrix SO. This results in an increased time between subsequent depolarization waves by ~ 10 s, thus reducing the frequency of oscillations. In contrast, increasing HP_stim_ from 1.5 × 10^–3^ to 2 × 10^–3^ decreased the time between depolarizations by about ~ 30 s, and therefore increased the propagating depolarization wave frequency. Since H_2_O_2_ diffused more readily to the mitochondria near the H_2_O_2_ source, those mitochondria accumulated H_2_O_2_ and thus also matrix SO more rapidly.

**Table 1:** Propagating Wave Depolarization Start Times (seconds).

		HP_stim_ (uM/ms)	
		1.5	2
**SO**_**stim**_** (pM/ms)**	1.2	231	154
		287	184
			207
			224
			245
			286
	1.4	221	147
		273	183
			220
			264
	1.5	218	139
		299	186
			238
			279

## Discussion

In this study we tested the hypothesis that H_2_O_2_ diffusion underlies the progression of mitochondrial network failure. To do so, we used a computational model of a mitochondrial network. The model included a detailed representation of the ROS scavenging system, ROS production from both intrinsic origins and externally induced alterations, and ROS diffusion between mitochondria. We regionally stimulated the network with ROS and observed the network behavior with and without H_2_O_2_ diffusion. We found that H_2_O_2_ diffusion was necessary to reproduce the mitochondrial network behavior observed in oxidative stress experiments. H_2_O_2_ diffused from the stimulus region and gradually depleted the H_2_O_2_ scavenging capacity of the local mitochondria. Growth of the region of scavenging capacity depletion enabled growth of the region of H_2_O_2_ accumulation. These findings demonstrate that H_2_O_2_ is an important communicator of oxidative stress that can distribute stress throughout the network prior to any signs of network failure from $\Delta \Psi_{{\text{m}}}$ depolarizations.

### H_2_O_2_ communication and accumulation enables so accumulation and communication

Depending on the tissue and cell type, different specific ROS were believed to play a more significant role in underlying mitochondrial network failure. In a modeling study by Park et al^33^, the dominant key messenger molecule of a ROS signaling network transitioned from the ROS SO to H_2_O_2_ as the distance between mitochondria increased. Thus, H_2_O_2_, with its improved diffusivity and longer lifetime over SO, was believed to play a comparatively more significant role in neurons, where the spacing between mitochondria was large. In ventricular cells, where there is less space between mitochondria versus neuronal cells, the dominant key messenger molecule in the RIRR phenomenon was found to be SO^8,9^. Propagating RIRR, where ROS release of a mitochondrion induces ROS release of a neighboring mitochondrion, occurs in ventricular cells when SO emission from a mitochondrion triggers opening of the IMAC of a neighboring mitochondrion^9,34^. The resulting expulsions of stored SO and other anions also depolarize the mitochondria's $\Delta \Psi_{{\text{m}}}$, which is a mitochondrial failure. Thus, SO transport was believed to play the dominant role in producing mitochondrial network failure^2,9,34^. However, propagation of this RIRR process relies on SO transport between neighbors that is large enough to trigger another IMAC opening^27^. Any initial SO release dissipates in magnitude over distance according to the laws of diffusion, and thus continuation of this propagation requires regenerative SO release across the network.

A mitochondrial network where enough oxidative stress has accumulated, such that any triggering of SO release can cause a cascade of SO releases across the network, has been termed "mitochondrial criticality"^34^. However, a chicken-and-egg problem arises when assuming SO is responsible for bringing the network to criticality because propagation of SO releases depends on prior SO accumulation. A different communicator of oxidative stress was required to bring the network to criticality prior to mitochondrial depolarization. In agreement with the observation that widespread CM-DCF oxidation (primarily an indicator of H_2_O_2_) precedes the first cell-wide depolarization^2^, the present results show that H_2_O_2_ can function as this alternative communicator of oxidative stress. Further, since H_2_O_2_ diffusion was necessary to observe mitochondrial depolarizations across the network, H_2_O_2_ communication underlies SO accumulation. As H_2_O_2_ diffused and accumulated throughout the network, scavenging of SO via SOD was inhibited^35,36^, which enabled SO to accumulate within the mitochondria matrices to produce a "critical" network. Thus, H_2_O_2_ still plays an important role in cell types where SO was believed to be the most important ROS in governing mitochondrial network failure.

### Delay in network response to oxidative stress is from scavenging capacity depletion time course

While H_2_O_2_ diffusion is the mechanism for how oxidative stress can communicate and spread throughout the network prior to mitochondrial depolarizations, it is unknown how this form of oxidative stress determines the progression of the network failure behavior. Laser stimulus- induced oxidative stress in a small region of the mitochondrial network consistently shows a delay of more than a minute before a cell-wide depolarization is observed^9,27^. Previous mitochondrial network models have been unable to reproduce this delayed behavior. Some type of "counter" mechanism must exist for any time-invariant system to display a delay effect, where the counter is some pool that is depleted or accumulated over time. Consistent with the behavior of a relaxation oscillator, when the counter crosses a threshold, downstream effects are triggered. Given a steady input of ROS to the mitochondrial network, we looked for patterns of system quantities gradually accumulating or depleting.

The mitochondrial network within ventricular cells contains a complex scavenging system that has a cascaded topology^37^. SO, which is an intrinsic by-product of the processes of the electron transport chain and the production of ATP, is continuously reduced by SOD to produce H_2_O_2_^25^. H_2_O_2_ is then scavenged by GSH peroxidase and thioredoxin-dependent peroxiredoxins to prevent H_2_O_2_ accumulation^25^. Regeneration of reduced GSH and thioredoxin is accomplished through NADPH-driven reductases, while NADPH redox potential ultimately depends on mitochondrial intermediates provided by the Krebs cycle. Modeling helps to reveal the dynamics of these redox-coupled reactions, and our results demonstrated that, indeed, GSH and NADPH are gradually depleted over time and across the network, prior to observing mitochondrial depolarizations. Further, our simulations also displayed the delay in response of the network to oxidative stress. SO accumulation and therefore the sensitivity of the network to perturbation, are potentiated by H_2_O_2_ accumulation, which occurs prior to GSH and NADPH depletion. The encasement of the region of matrix SO accumulation within the regions of H_2_O_2_ accumulation (Fig. 6b) and the NADPH and GSH depletion regions (Fig. 5b), demonstrates the sequential cascaded topology of the scavenging system. Thus, the delay in response of the network is caused by the time it takes to deplete the NADPH and GSH reserves of the network, and simultaneously, to accumulate H_2_O_2_ and SO throughout the network. Our model is able to represent the evolution of a mitochondrial network gradually overwhelmed by oxidative stress.

### Modeling variations in mitochondrial network failure behavior

There are variations in the protocols for how oxidative stress is introduced to mitochondrial networks. The common methods of experiments include bathing the network in exogenous ROS solution^27^, providing a laser stimulus that locally produces ROS^9^, metabolically stressing the system via ischemia/reperfusion protocols^38,39^, or by reducing scavenging capacity of the system via targeted inhibitory chemicals^17,40^. Each method affects the distribution of oxidative stress and the overall response of the network. Models have been developed to try study these systems and reproduce some of the behavior of these experiments. A previous model by Yang^28^ examined how the short range effects of SO and the longer range effects of H_2_O_2_ could participate to independently activate IMAC and PTP, respectively; however, the impact of H_2_O_2_ accumulation (with consequent NADPH and GSH depletion) on the SO triggering mechanism was not explored. In a subsequent model from Nivala et al.^3^, SO-mediated IMAC activation was modeled as a stochastic process that caused local propagations that could, depending on SO levels, sum to produce propagating waves of ∆Ψ_m_ depolarization, but the effect of H_2_O_2_ was not included. Also, models from our lab^27^ have been used to study propagation mechanisms during oxidatively stressed conditions. These models, however, do not capture the delay in response of the network to oxidative stress or demonstrate the relationship between H_2_O_2_ diffusion, the SO accumulation, or the ultrasensitive nature of the ∆Ψ_m_ oscillations. By varying the ratio of H_2_O_2_ and SO, our model shows that altering the H_2_O_2_ stimulus modulates the growth rate of the oscillatory region, whereas altering the SO stimulus alters the frequency of oscillations. Full validation of the model will require accurate measurements of the different distributions of ROS across the network of a cell and examination of the impact of network topology on propagations and synchronization. Nevertheless, we believe that the specific dependencies of H_2_O_2_ and SO on long- and short-range coherent behavior, respectively, should remain.

The present model supports the feasibility of long range ROS-dependent communication in the mitochondrial network, but does not exclude the possibility of direct physical communication between mitochondria, for example, through nanotunnel structures^41^. However, such structures have only been shown to span distances of ~ 1–5 µm (roughly 1–2 sarcomeres) within cardiomyocytes, which could not account for synchronization of oscillations over the entire length of the adult heart cell (> 100 µm). Glancy and Balaban have proposed connections over even larger distances^11^, however our model is based on experimental observations that mitochondria in adult myocytes predominantly behave independently^8,20,42^. Assuming mitochondria are connected in a reticulum implies strong electrical coupling that would alter the sensitivity of the network and change the dynamics/spatial patterns of the depolarization waves. A further modeling study would be needed to properly characterize these changes.

Further study is also needed to see how the effects of H_2_O_2_ communication are important in experiments with ischemia/reperfusion protocols, and how this network behavior scales up in tissue and in the whole heart. We hypothesize that the high mobility of H_2_O_2_ is important in distributing oxidative stress at the tissue and whole heart levels and may be important for producing the heterogenous distributions of network oscillation phases that may play a role in arrhythmogenesis. Incorporating our network model into a cardiac tissue model can be used to investigate these unknowns.

## Methods

### Computational model

The model developed here uses as its basis the mitochondrial network model by Zhou et al.^27^, which describes self-organized $\Delta \Psi_{{\text{m}}}$ oscillations mediated by Ca^2+^-independent RIRR via IMAC. Currently, there is no precise identification of the molecular structure of IMAC, so like previous models^25,27^, IMAC is a model assumption. The model does not incorporate Ca^2+^-dependent activation of PTP, which can be sensitized to open by higher levels of sustained oxidative stress^40^. The model represents the mitochondrial network as a collection of closely apposed nodes (mitochondria), with inter-connections represented by SO diffusion. Each individual mitochondrion is represented by the model of Cortassa et al.^25^, where ordinary differential equations describe the evolution of the mitochondrial matrix and intermitochondrial space. The model includes descriptions of the TCA cycle, oxidative phosphorylation, Ca^2+^ handling, SO production, SO transport via IMAC from matrix to intermitochondrial space, and both SO and H_2_O_2_ scavenging.

We added new components to the single mitochondrion model (Fig. 2a) to enable us to better represent ROS scavenging. First, we added a representation of the mitochondrial matrix scavenger superoxide dismutase (SOD) to address its possible role in H_2_O_2_ dynamics, as matrix SO is converted to H_2_O_2_. Second, the H_2_O_2_ scavenging system was further developed to include the thioredoxin system, as described by Kembro et al^37^, since it supplements the glutathione (GSH) system in scavenging H_2_O_2_ and thus affects H_2_O_2_ dynamics. Third, we incorporated parameterized formulations of nicotinamide adenine dinucleotide phosphate (NADPH) dynamics. NADPH drives the production of GSH and thioredoxin and thus plays a role in maintaining H_2_O_2_ scavenging capacity. We parameterized the size of the NADPH pool and its replenishment rate to set limits on the transient and steady state capacity for maintaining a functioning H_2_O_2_ scavenging system. Finally, we removed the representation of catalase to highlight the effects of the energetically driven H_2_O_2_ scavenging systems, because the catalase content in the cardiac ventricular myocyte is low^43^, and because local catalase concentrations within the different cell compartments are not known.

In addition to further developing ROS scavenging, we redefined some and added other ROS production components to the single mitochondrion model. In their model, Cortassa et al. represented the basal SO produced from the electron transport chain as a function of oxygen consumed. This model mechanism is a positive feedback loop where SO production increases as SO accumulates within the matrix. However, to control for the contributions of SO production rate, we redefined the mitochondrial intrinsic SO production rate to be parameterized as SO_base_ (7.5 × 10^−6^ mM ms^−1^). By doing so, we excluded electron transport chain driven oscillatory pressure and instead highlighted the pressure due to scavenging dynamics. We also added the two parameters for ROS stimulation, HP_stim_ and SO_stim_, which are the respective H_2_O_2_ and SO production rates during any given stimulation protocol.

Completing model development, we added H_2_O_2_ diffusion between mitochondria to enable H_2_O_2_ to act as a communicator of oxidative stress across the network (Fig. 2b). We used a higher diffusion coefficient for H_2_O_2_, as compared to SO, to represent the higher diffusibility of H_2_O_2_ over SO between mitochondria^44,45^. Similar to Zhou et al.^27^, mitochondria in our network model were arranged in a line and spaced 1um apart, the average distance measured experimentally in cells^46^. A total of 100 mitochondria were represented in the model. This number was determined iteratively to be sufficiently large to observe all the morphological changes in the depolarization patterns of the network while simultaneously minimizing computational expense. Similar to Zhou et al.^27^, we assumed a no flux boundary condition for SO diffusion and reapplied this assumption for H_2_O_2_ diffusion. Previous mitochondrial network models had assumed the same boundary condition for H_2_O_2_ diffusion^28^.

### Oxidative stress stimulation protocol

To test our hypothesis, we simulated an oxidatively stressed mitochondrial network for 300 s. This is a typical time span to observe network failure progression in oxidative stress experiments^9,27^. We introduced oxidative stress to the network by stimulating a small group of three neighboring mitochondria in the network (Fig. 3a). Stimulation of these mitochondria was represented by assigning a value to their SO and H_2_O_2_ production rate (SO_stim_ and HP_stim_ respectively). The value of the stimulus was maintained constant during the entire simulation duration (300 s). To be able to compare simulation results to experimental, our stimulation protocol mimicked the stimulation protocol used in laser flash experiments, where a brief (~ 500 ms) localized laser flash induces sustained ROS production in a localized region of the network^9^. The central mitochondrion in the group of three was assigned the largest SO_stim_ of 5 × 10^−5^ mM ms^−1^ (Fig. 3a), which was iteratively determined to be the smallest value needed to induce a permanent depolarization in that mitochondrion. The remaining 2 stimulated mitochondria were assigned a smaller SO_stim_ of 1.4 × 10^−5^ mM ms^−1^ to represent the reduced laser intensity from being located at the edge of the laser flash region. Production of H_2_O_2_ was represented by assigning the center mitochondrion's HP_stim_ equal to 2 × 10^−3^ mM ms^−1^. To determine the effect of H_2_O_2_ transport on the progression of mitochondrial network failure, we performed this stimulation protocol with and without H_2_O_2_ diffusion. Further, to explore the effect of different ROS stimulus makeups, we repeated these simulations with different HP_stim_ rates of 1 × 10^−3^ mM ms^−1^, 1.5 × 10^−3^ mM ms^−1^, and 2 × 10^−3^ mM ms^−1^, and different SO_stim_ rates of 12 × 10^−6^ mM ms^−1^, 14 × 10^−6^ mM ms^−1^, and 16 × 10^−6^ mM ms^−1^. These alterations to the previous stimulus rates were iteratively determined to be large enough to demonstrate a change in network behavior.

### Detail of mitochondrial model modifications

The following describes the details of the model additions and changes from previous models. Listed variable names correspond to the variable names in the model equations file.

We made changes to the Zhou model^27^ to better represent ROS scavenging. First, we added a representation of the mitochondrial matrix SOD. We reused the same formulation of the scavenging rate via intermitochondrial SOD (VSOD_i) from the Cortassa model^25^ for the matrix SOD scavenging rate (VSOD_m). The concentration of matrix SOD, compared to intermitochondrial SOD, was reduced from 1.43 uM to 300 nM to represent the reduced availability of SOD in the matrix due to the differences in volume of the two spaces. Second, we updated the parameters of the glutathione based H_2_O_2_ scavenging system. The concentration for glutathione peroxidase (E__GPX_T), the concentration for glutathione reductase (E__GR_T), the K constants for GSSG (K_GSSG_M) and NADPH (K__NADPH), and total glutathione pool (G_tot) was taken from the 2013 Gauthier paper^47^, Phi_2, and k_GR were taken from the other 2013 Gauthier paper^48^ because the first paper’s^47^ supplement values appeared to have a unit conversion error. Third, we included the thioredoxin/peroxiredoxin based H_2_O_2_ scavenging system. The equations for this component was taken from the first 2013 Gauthier paper’s supplement^47^. Similar to the glutathione changes, the values for Phi were taken from the other 2013 Gauthier supplement^48^. Finally, we parameterized the size of the NADPH pool, using a starting capacity value of 12 mM and a replenishment rate of 7.5 nM/ms (NADPH_gen).

In addition to SO diffusion, we added H_2_O_2_ diffusion. The resulting equations for ROS flux were:

$$ \begin{aligned} {\text{neighborSO}}\_{\text{flux}} & = \left( {{\text{SOi}}\_{\text{left}} + {\text{SOi}}\_{\text{right}} - {2}*{\text{SOi}}\_{\text{self}}} \right)/{\text{C}}\_{\text{SO}} \\ {\text{neighborHP}}\_{\text{flux}} & = \left( {{\text{HP}}\_{\text{left}} + {\text{HP}}\_{\text{right}} - {2}*{\text{HP}}\_{\text{self}}} \right)/{\text{C}}\_{\text{HP}} \\ \end{aligned} $$

SOi_(left|right) was the SO concentration of the neighbors. HP_(left|right) was the H_2_O_2_ concentration of the neighboring mitochondria. The diffusion coefficient for SO (C_SO) was unchanged from the Zhou model^27^ and 4 mM/ms/um was used for the diffusion coefficient for H_2_O_2_ (C_HP).

With the changes just described, the resulting differential equations for the affected state variables were:

$$ \begin{aligned} & {\text{dSOm}}/{\text{dt}} = {\text{SO}}\_{\text{stim}} - {\text{VSOD}}\_{\text{m}} - {\text{IMAC}}\_{\text{flux}} \\ & {\text{dSOi}}/{\text{dt}} = {\text{IMAC}}\_{\text{flux}} - {\text{VSOD}}\_{\text{i}} + {\text{neighborSO}}\_{\text{flux}} \\ & {\text{dNADPH}}/{\text{dt}} = {\text{NADPH}}\_{\text{gen}} - {\text{VGR}} - {\text{VTRXR}} \\ & {\text{dH2O2}}/{\text{dt}} = {\text{HP}}\_{\text{stim}} + {\text{VSOD}}\_{\text{m}} + {\text{VSOD}} - {\text{VGPX}} - {\text{VPRX}} + {\text{neighborHP}}\_{\text{flux}} \\ \end{aligned} $$

VGR is the rate of production of glutathione from glutathione reductase using NADPH and the precursor GSSG. Similarly, VTRXR is for replenishment of thioredoxin. VGPX and VPRX are the H_2_O_2_ scavenging rates from the scavenging systems governed by GSH and thioredoxin respectively. The constants used for VGR in the Zhou et al. model^27^ were updated to the more recent values in Gauthier et al. ^47,48^ with the changes for the constants described above. VTRXR and VPRX was also added from the Gauthier models.

### Computational methods

The nonlinear system of partial differential equations describing ROS communication between nodes was spatially discretized using the finite difference method. The aggregated ODEs of all the nodes were numerically integrated using the solver CVODE, a multistep stiff ODE solver that uses a banded backward differentiation formula method and a direct linear solver to implement newton iteration. A maximum time step of 0.1 ms was used to stably simulate the model on a desktop computer. Model source code is freely available for download on github: https://github.com/bmillare/RIRR-propagation-sensitivity-model

## References

[1] Sasaki N, Sato T, Marbán E, O’Rourke B (2001). ATP consumption by uncoupled mitochondria activates sarcolemmal K(ATP) channels in cardiac myocytes. Am. J. Physiol. Heart Circ. Physiol..

[2] Aon MA, Cortassa S, O’Rourke B (2004). Percolation and criticality in a mitochondrial network. Proc. Natl. Acad. Sci. U. S. A..

[3] Nivala M, Korge P, Nivala M, Weiss JN, Qu Z (2011). Linking flickering to waves and whole-cell oscillations in a mitochondrial network model. Biophys. J..

[4] Stanley WC, Recchia FA, Lopaschuk GD (2005). Myocardial substrate metabolism in the normal and failing heart. Physiol. Rev..

[5] Saks V (2006). Cardiac system bioenergetics: metabolic basis of the Frank-Starling law. J. Physiol..

[6] Ichas F, Jouaville LS, Mazat J-P (1997). Mitochondria are excitable organelles capable of generating and conveying electrical and calcium signals. Cell.

[7] Romashko DN, Marban E, O’Rourke B (1998). Subcellular metabolic transients and mitochondrial redox waves in heart cells. Proc. Natl. Acad. Sci..

[8] Zorov DB, Filburn CR, Klotz LO, Zweier JL, Sollott SJ (2000). Reactive oxygen species (ROS)-induced ROS release: a new phenomenon accompanying induction of the mitochondrial permeability transition in cardiac myocytes. J. Exp. Med..

[9] Aon MA, Cortassa S, Marbán E, O’Rourke B (2003). Synchronized whole cell oscillations in mitochondrial metabolism triggered by a local release of reactive oxygen species in cardiac myocytes. J. Biol. Chem..

[10] Amchenkova AA, Bakeeva LE, Chentsov YS, Skulachev VP, Zorov DB (1988). Coupling membranes as energy-transmitting cables. I. Filamentous mitochondria in fibroblasts and mitochondrial clusters in cardiomyocytes. J. Cell Biol..

[11] Glancy B (2015). Mitochondrial reticulum for cellular energy distribution in muscle. Nature.

[12] Crucitti P, Latora V, Marchiori M (2004). Model for cascading failures in complex networks. Phys Rev E Stat Nonlin Soft Matter Phys..

[13] Zhou L (2014). Effects of regional mitochondrial depolarization on electrical propagation: Implications for arrhythmogenesis. Circ. Arrhythmia Electrophysiol..

[14] Akar FG, Aon MA, Tomaselli GF, O’Rourke B (2005). The mitochondrial origin of postischemic arrhythmias. J. Clin. Invest..

[15] Jeong E-M (2012). Metabolic stress, reactive oxygen species, and arrhythmia. J. Mol. Cell. Cardiol..

[16] Dey S, DeMazumder D, Sidor A, Foster DB, O’Rourke B (2018). Mitochondrial ROS Drive Sudden Cardiac Death and Chronic Proteome Remodeling in Heart Failure.

[17] Slodzinski MK, Aon MA, O’Rourke B (2008). Glutathione oxidation as a trigger of mitochondrial depolarization and oscillation in intact hearts. J. Mol. Cell. Cardiol..

[18] Brown DA, O’Rourke B (2010). Cardiac mitochondria and arrhythmias. Cardiovasc. Res..

[19] Lyon AR (2010). Optical imaging of mitochondrial function uncovers actively propagating waves of mitochondrial membrane potential collapse across intact heart. J. Mol. Cell. Cardiol..

[20] Hou T, Wang X, Ma Q, Cheng H (2014). Mitochondrial flashes: new insights into mitochondrial ROS signalling and beyond. J. Physiol..

[21] Wang W (2008). Superoxide flashes in single Mitochondria. Cell.

[22] Lu X, Kwong J, Molkentin JD, Bers DM (2016). Individual cardiac mitochondria undergo rare transient permeability transition pore openings. Circ. Res..

[23] Zhou L, O’Rourke B (2012). Cardiac mitochondrial network excitability: insights from computational analysis. AJP Hear. Circ. Physiol..

[24] Kusama Y, Bernier M, Hearse DJ (1989). Singlet oxygen-induced arrhythmias Dose- and light-response studies for photoactivation of rose bengal in the rat heart. Circulation.

[25] Cortassa S, Aon MA, Winslow RL, O’Rourke BA (2004). mitochondrial oscillator dependent on reactive oxygen species. Biophys. J..

[26] Zorov DB, Juhaszova M, Sollott SJ (2014). Mitochondrial reactive oxygen species (ROS) and ROS-induced ROS release. Physiol. Rev..

[27] Zhou L (2010). A reaction-diffusion model of ROS-induced ROS release in a mitochondrial network. PLoS Comput. Biol..

[28] Yang L, Korge P, Weiss JN, Qu Z (2010). Mitochondrial oscillations and waves in cardiac myocytes: Insights from computational models. Biophys. J..

[29] Kurz FT, Derungs T, Aon MA, O’Rourke B, Armoundas AA (2015). Mitochondrial networks in cardiac myocytes reveal dynamic coupling behavior. Biophys. J..

[30] Kurz FT, Aon MA, O’Rourke B, Armoundas AA (2014). Cardiac mitochondria exhibit dynamic functional clustering. Front. Physiol..

[31] Kurz FT, Aon MA, O’Rourke B, Armoundas AA (2010). Spatio-temporal oscillations of individual mitochondria in cardiac myocytes reveal modulation of synchronized mitochondrial clusters. Proc. Natl. Acad. Sci. USA.

[32] Kurz FT, Aon MA, O’Rourke B, Armoundas AA (2010). Wavelet analysis reveals heterogeneous time-dependent oscillations of individual mitochondria. Am. J. Physiol. Heart Circ. Physiol..

[33] Park J, Lee J, Choi C (2011). Mitochondrial network determines intracellular ROS dynamics and sensitivity to oxidative stress through switching inter-mitochondrial messengers. PLoS ONE.

[34] Aon MA, Cortassa S, Akar FG, O’Rourke B (2006). Mitochondrial criticality: a new concept at the turning point of life or death. Biochim. Biophys. Acta Mol. Basis Dis..

[35] Bray RC (1974). Reduction and inactivation of superoxide dismutase by hydrogen peroxide. Biochem. J..

[36] Liochev SI, Fridovich I (2002). Copper, zinc superoxide dismutase and H2O2: effects of bicarbonate on inactivation and oxidations of Nadph and Urate, and on consumption of H2O2. J. Biol. Chem..

[37] Kembro JM, Aon MA, Winslow RL, O’Rourke B, Cortassa S (2013). Integrating mitochondrial energetics, redox and ROS metabolic networks: a two-compartment model. Biophys. J..

[38] Brady NR, Hamacher-Brady A, Westerhoff HV, Gottlieb RA (2006). A wave of reactive oxygen species (ROS)-induced ROS release in a sea of excitable mitochondria. Antioxid. Redox Signal..

[39] Solhjoo S, O’Rourke B, Manuscript A, Structures T (2015). Mitochondrial instability during regional ischemia-reperfusion underlies arrhythmias in monolayers of cardiomyocytes. J Mol Cell Cardiol..

[40] Aon MA, Cortassa S, Maack C, O’Rourke B (2007). Sequential opening of mitochondrial ion channels as a function of glutathione redox thiol status. J. Biol. Chem..

[41] Lavorato M (2017). Increased mitochondrial nanotunneling activity, induced by calcium imbalance, affects intermitochondrial matrix exchanges. Proc. Natl. Acad. Sci..

[42] Boyman L (2014). Calcium movement in cardiac mitochondria. Biophys. J..

[43] Chance B, Sies H, Boveris A (1979). Hydroperoxide metabolism in mammalian organs. Physiol. Rev..

[44] Han D, Antunes F, Canali R, Rettori D, Cadenas E (2003). Voltage-dependent anion channels control the release of the superoxide anion from mitochondria to cytosol. J. Biol. Chem..

[45] Bienert GP, Chaumont F (2014). Aquaporin-facilitated transmembrane diffusion of hydrogen peroxide. Biochim. Biophys. Acta Gen. Subj..

[46] Birkedal R, Shiels HA, Vendelin M (2006). Three-dimensional mitochondrial arrangement in ventricular myocytes: from chaos to order. Am. J. Physiol. Cell Physiol..

[47] Gauthier LD, Greenstein JL, Cortassa S, O’Rourke B, Winslow RL (2013). A Computational model of reactive oxygen species and redox balance in cardiac mitochondria. Biophys. J..

[48] Gauthier LD, Greenstein JL, O’Rourke B, Winslow RL (2013). An integrated mitochondrial ROS production and scavenging model: implications for heart failure. Biophys. J..

